# Triterpenoid saponins from the roots of *Acanthophyllum gypsophiloides* Regel

**DOI:** 10.3762/bjoc.8.87

**Published:** 2012-05-23

**Authors:** Elena A Khatuntseva, Vladimir M Men’shov, Alexander S Shashkov, Yury E Tsvetkov, Rodion N Stepanenko, Raymonda Ya Vlasenko, Elvira E Shults, Genrikh A Tolstikov, Tatjana G Tolstikova, Dimitri S Baev, Vasiliy A Kaledin, Nelli A Popova, Valeriy P Nikolin, Pavel P Laktionov, Anna V Cherepanova, Tatiana V Kulakovskaya, Ekaterina V Kulakovskaya, Nikolay E Nifantiev

**Affiliations:** 1 Laboratory of Glycoconjugate Chemistry, N. D. Zelinsky Institute of Organic Chemistry, Russian Academy of Sciences, Leninsky prospect 47, 119991 Moscow, Russian Federation; 2 Laboratory of NMR spectroscopy, N. D. Zelinsky Institute of Organic Chemistry, Russian Academy of Sciences, Leninsky prospect 47, 119991 Moscow, Russian Federation; 3 Institute of Immunology, Ministry of Health and Social Development of Russian Federation, Kashirskoe Chausseе, 24/2, 115478 Moscow, Russian Federation; 4 Laboratory of Pharmacological Researches N. N. Vorozhtsov Novosibirsk Institute of Organic Chemistry, Siberian Branch of the Russian Academy of Sciences, prospect Acad. Lavrent’eva, 9, 630090 Novosibirsk, Russian Federation; 5 Institute of Cytology and Genetics Siberian Branch of the Russian Academy of Sciences, 10 prospect Acad. Lavrent’eva, 630090 Novosibirsk, Russian Federation; 6 Institute of Chemical Biology and Fundamental Medicine, Siberian Branch of the Russian Academy of Sciences, 8 prospect Acad. Lavrent’eva, 630090 Novosibirsk, Russian Federation; 7 G. K. Skryabin Institute of Biochemistry and Physiology of Microorganisms, Russian Academy of Sciences, 142290 Pushchino, Moscow region, Russian Federation

**Keywords:** *Acanthophyllum gypsophiloides*, adjuvant, hemolysis, NMR, saponin, structure

## Abstract

Two new triterpenoid saponins **1** and **2** were isolated from the methanol extract of the roots of *Acanthophyllum gypsophiloides* Regel*.* These saponins have quillaic acid or gypsogenin moieties as an aglycon, and both bear similar sets of two oligosaccharide chains, which are 3-*O*-linked to the triterpenoid part trisaccharide α-L-Ara*p*-(1→3)-[α-D-Gal*p*-(1→2)]-β-D-Glc*p*A and pentasaccharide β-D-Xyl*p*-(1→3)-β-D-Xyl*p*-(1→3)-α-L-Rha*p*-(1→2)-[β-D-Qui*p*-(1→4)]-β-D-Fuc*p* connected through an ester linkage to C-28. The structures of the obtained saponins were elucidated by a combination of mass spectrometry and 2D NMR spectroscopy. A study of acute toxicity, hemolytic, anti-inflammatory, immunoadjuvant and antifungal activity was carried out. Both saponins **1** and **2** were shown to exhibit immunoadjuvant properties within the vaccine composition with keyhole limpet hemocyanin-based immunogen. The availability of saponins **1** and **2** as individual pure compounds from the extract of the roots of *A. gypsophiloides* makes it a prospective source of immunoactive agents.

## Introduction

Triterpenoid saponins [1] occur in many plant species and have a diverse range of properties [2]. Nowadays, a steadily growing number of publications [3–10] are aimed at research on saponins as potential adjuvants, with an urgent demand due to the fast development of immunotherapy methods. Among the most efficient saponin adjuvants are the components of a complex mixture of triterpenoids extracted from the bark of *Quillaja saponaria* Molina, which are used in veterinary vaccines [4]. One of the best known products of this origin is the less toxic and more stable fraction QS-21 [8], which is included now into a vast range of pilot vaccine compositions against viral infections [11–13] and cancer [14–17].

Meanwhile, the search for abundant, nontoxic, stable and individual saponin adjuvants is still urgent. This explains the enormous interest in investigations into saponins, particularly concerning the study of the relationships between their structure, adjuvant activity and toxicity [5,7]. In this paper we report the isolation and structural assessment of two saponins from the roots of *A. gypsophiloides* Rgl. (Turkestan soap root) with the further investigation of their toxicity, hemolytic activity, anti-inflammatory, antifungal and adjuvant properties. The roots of *A. gypsophiloides* Rgl. are an easily available raw material, which was reported [18] to comprise a saponin with a structure that is close to that of the extremely efficient adjuvant QS-21. *A. gypsophiloides* Rgl. is a member of the genus *Caryophyllaceae* (for other saponins see [19–22]), which is widely spread in mountain areas of central Asia. The crude saponin-containing fraction from the roots of *A. gypsophiloides* Rgl. has been known to be an excellent foaming agent for food and nutrition industry, and its composition has previously been under investigation [18].

## Results and Discussion

**Figure 1:**
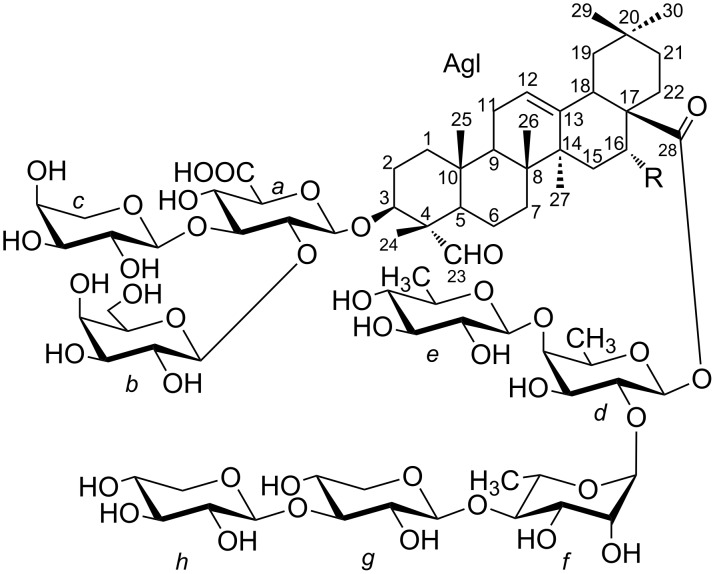
Saponins from *A. gypsophiloides* **1**, R = OH and **2**, R = H.

The methanolic extract of the dried powdered roots of *A. gypsophiloides* was concentrated, and the crude mixture of saponins was precipitated from methanol by the addition of acetone and subjected to reversed-phase С18 HPLC. Compounds **1** and **2** (Figure 1) were isolated as white amorphous powders. Compound **1** exhibited in the HRMS (ESI) the [M + Na]^+^ peak at *m*/*z* 1681.7071, indicating a molecular weight compatible with the molecular formula C_75_H_118_O_40_. Compound **2** exhibited the [M + Na]^+^ peak at *m*/*z* 1665.7181, consistent with the molecular formula С_75_H_118_O_39_. GLC analysis of the acetylated (*S*)-2-octyl glycosides derived after full acid hydrolysis of compound **1** revealed the presence of D-galactose (D-Gal), L-arabinose (L-Ara), 6-deoxy-D-glucose (D-Qui), D-xylose (D-Xyl), L-rhamnose (L-Rha), D-fucose (D-Fuc), and D-glucuronic acid (D-GlcA). Similar investigation of compound **2** revealed the same sugar composition as for compound **1**.

The structures of both compounds **1** and **2** were confirmed on the basis of their ^1^H NMR, ^13^C NMR, APT, COSY, TOCSY, ROESY, HSQC, and HMBC spectra. In accordance with the earlier reports [18] on structures of saponins from *A. gypsophiloides*, the aglycons of compound **1** and **2** were supposed to comprise quillaic acid (16-α-hydroxygypsogenin) and gypsogenin, respectively. This assumption was in good agreement with the detection of characteristic signals for six methyl groups in the ^1^H (Table 1) and ^13^C NMR (Table 2) spectra of **1** and **2**. Furthermore, the presence of these aglycons was unambiguously confirmed by the good agreement between ^13^C NMR shifts of aglycon moieties of **1** and **2** and signals of aglycons for described bidesmosides comprising quillaic acid [21] and gypsogenin [21].

**Table 1:** ^1^H and ^13^С NMR data (δ, ppm) of the triterpene units of compounds **1** and **2** (500 MHz, pyridine-*d*_5_/D_2_O 1:1).*^a^*

Comp.	C-1	C-2	C-3	C-4	C-5	C-6	C-7	C-8	C-9	C-10	C-11	C-12	C-13	C-14	C-15
	*H-1*	*H-2*	*H-3*		*H-5*	*H-6*	*H-7*		*H-9*		*H-11*	*H-12*			*H-15*

**1**	38.3	25.2	85.3	55.9	48.3	20.7	32.8	40.4	47.0	36.3	23.9	122.7	144.1	42.2	35.9
	*1.53*	*2.28*	*4.06*		*1.37*	*1.40*	*1.62*		*1.75*		*1.91*	*5.37*			*2.04*
	*0.91*	*1.97*				*1.01*	*1.49*				*1.86*				*1.89*
**2**	38.2	25.1	85.4	56.0	48.2	20.8	32.6	40.2	47.8	36.3	23.8	122.8	144.1	42.5	28.7
	*1.51*	*2.29*	*4.10*		*1.43*	*1.43*	*1.62*		*1.66*		*1.87*	*5.37*			*1.81*
	*0.94*	*1.97*				*1.08*	*1.48*				*1.82*				*1.42*

Comp.	C-16	C-17	C-18	C-19	C-20	C-21	C-22	C-23	C-24	C-25	C-26	C-27	C-28	C-29	C-30
	*H-16*		*H-18*	*H-19*		*H-21*	*H-22*	*H-23*	*H-24*	*H-25*	*H-26*	*H-27*		*H-29*	*H-30*

**1**	73.9	47.9	41.6	47.4	29.3	35.8	31.5	211.6	10.7	16.0	17.6	27.3	177.1	33.1	24.6
	*5.01*		*3.27*	*2.57*		*2.19*	*2.28*	*9.71*	*1.43*	*0.88*	*0.96*	*1.68*		*0.94*	*0.96*
				*1.24*		*1.26*	*2.04*								
**2**	23.3	47.9	42.1	46.4	30.8	33.9	32.4	211.5	10.7	15.8	17.5	26.1	176.4	33.2	23.7
	*2.05*		*2.99*	*1.68*		*1.25*	*1.82*	*9.63*	*1.43*	*0.85*	*0.92*	*1.24*		*0.93*	*0.85*
	*1.75*			*1.17*		*1.14*	*1.66*								

^a1^H NMR chemical shifts are italicized.

**Table 2:** ^1^H and ^13^С NMR data (δ, ppm; *J*, Hz) for carbohydrate units of compounds **1** and **2** (500 MHz, pyridine-*d*_5_/D_2_O 1:1).

Units, atoms	**1**			**2**	
			
	δ_C_	δ_H_ (*J*)		δ_C_	δ_H_ (*J*)

→2,3)-GlcA (*a*)					
1	103.4	4.83, d (7.8)		103.4	4.82, d (7.3)
2	77.7	4.26		77.7	4.27
3	85.0	4.30		85.0	4.31
4	71.6	4.16		71.6	4.17
5	77.7	4.26		77.7	4.27
6	175.2			175.2	

Gal (*b*)					
1	103.2	5.33, d (7.7)		103.2	5.33, d (7.5)
2	72.8	4.14		72.8	4.14
3	74.4	4.09		74.5	4.08
4	70.3	4.31		70.3	4.31
5	76.5	3.97		76.5	3.97
6(a, b)	62.2	4.33, 4.17		62.1	4.35, 4.17

Ara (*c*)					
1	104.0	5.16, d (7.5)		104.0	5.17, d (7.5)
2	72.4	4.23		72.4	4.23
3	73.7	4.12		73.8	4.12
4	69.3	4.28		69.4	4.28
5(a, b)	67.2	4.34, 3.95		67.2	4.34, 3.95

→2,4)-Fuc (*d*)					
1	94.4	5.78, d (8.1)		94.5	5.80, d (8.1)
2	74.6	4.43		75.1	4.41
3	76.3	4.20		76.0	4.19
4	83.2	4.12		83.0	4.12
5	71.9	4.03		71.8	4.02
6	17.1	1.52		17.1	1.52

Qui (*e*)					
1	105.6	4.92, d (7.8)		105.6	4.92, d (7.8)
2	75.6	3.81		75.6	3.80
3	77.0	3.99		77.1	4.00
4	76.1	3.53		76.1	3.53
5	72.9	3.69		72.9	3.70
6	18.2	1.51		18.2	1.51

→4)-Rha (*f*)					
1	101.2	6.01 s (<1)		101.2	5.97 s (<1)
2	71.1	4.62		71.1	4.62
3	71.8	4.41		71.8	4.43
4	83.7	4.15		83.7	4.18
5	68.3	4.26		68.7	4.28
6	18.3	1.65		18.4	1.68

→3)-Xyl (*g*)					
1	106.1	5.06, d (8.5)		105.9	5.09, d (7.7)
2	74.7	3.92		74.7	3.91
3	86.5	4.02		86.4	4.01
4	68.8	3.99		68.8	4.00
5(a, b)	66.2	4.18, 3.58		66.2	4.18, 3.58

Xyl (*h*)					
1	104.9	5.03, d (8.8)		104.9	5.04, d (7.6)
2	74.7	3.92		74.7	3.91
3	77.0	4.01		77.1	4.01
4	70.2	4.09		70.2	4.09
5(a, b)	66.5	4.30, 3.69		66.5	4.30, 3.68

**Figure 2:**
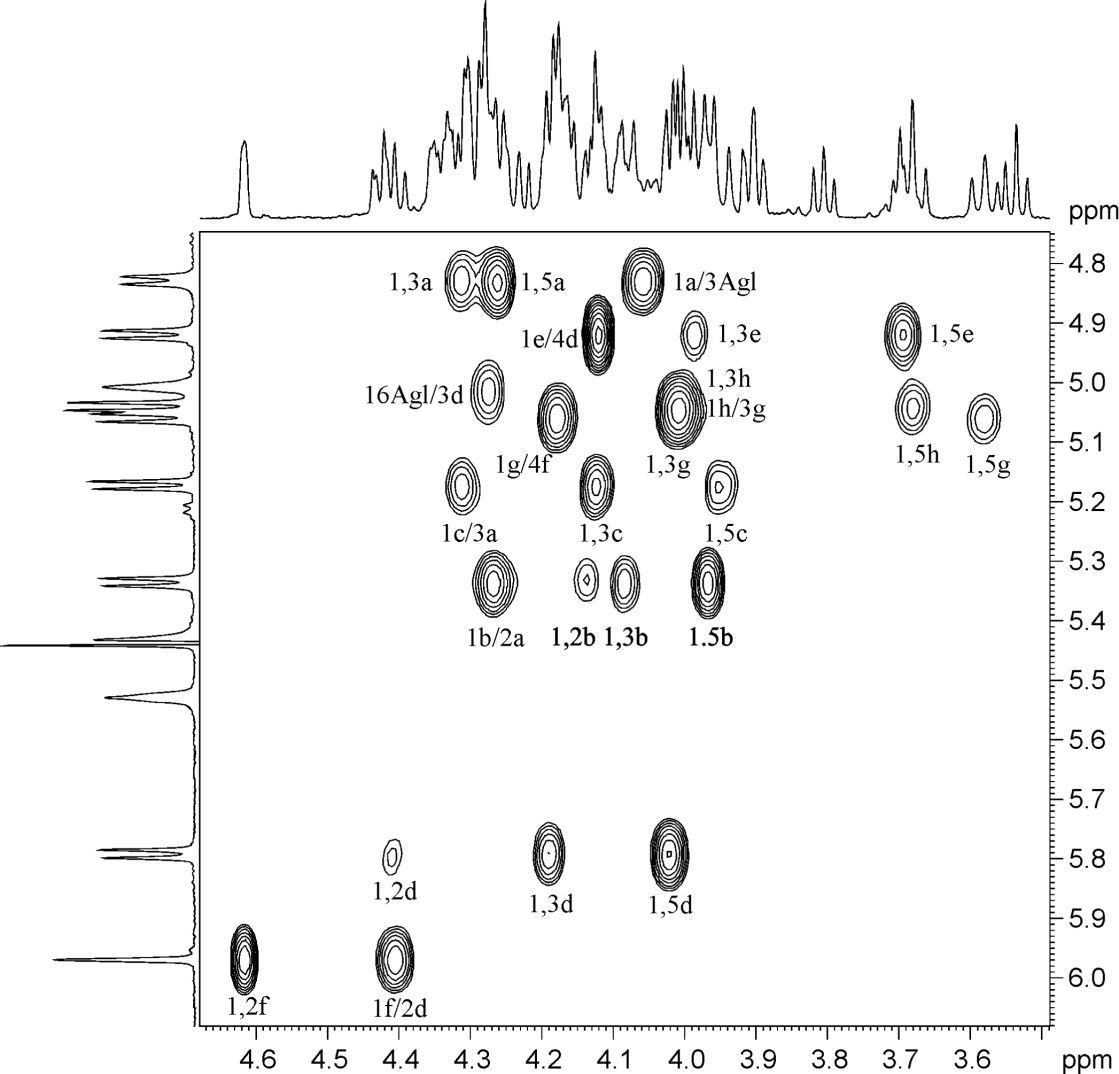
Part of a 2D ROESY spectrum of compound **1**. The corresponding parts of the ^1^H NMR spectrum are shown along the axes. Arabic numerals refer to atoms in sugar residues denoted by letters, as shown for compounds **1** and **2**. Slashes are used for the designation of inter-residual interactions.

**Figure 3:**
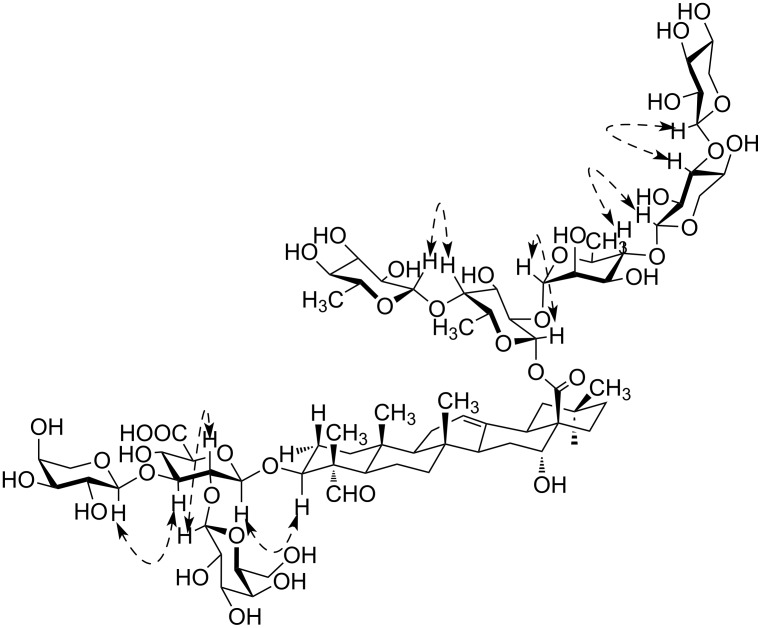
Key ROESY (dashed line) correlations for compound **1**.

**Figure 4:**
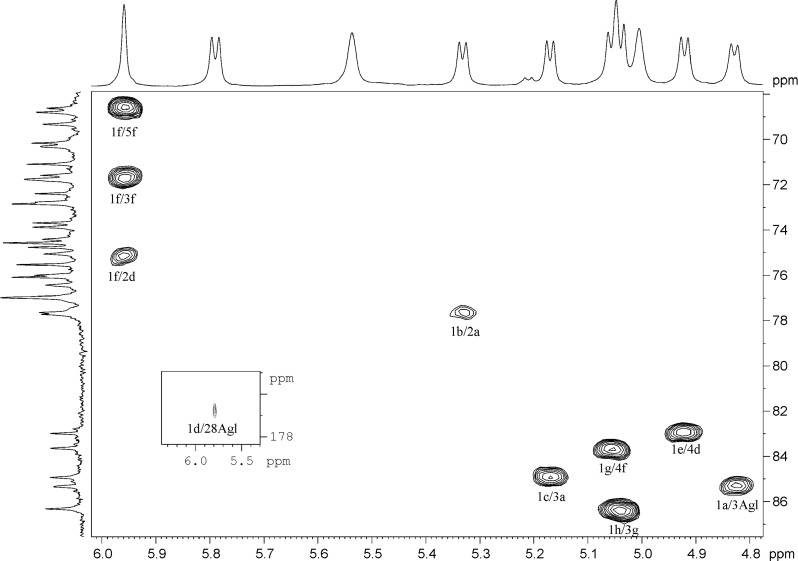
Part of the HMBC spectrum of compound **1**. ^1^H and ^13^C NMR spectra are shown along the horizontal and vertical axes, respectively. Arabic numerals before a slash refer to protons and after a slash refer to carbons in sugar residues denoted by letters, as shown for compounds **1** and **2**.

Analysis of COSY and TOCSY spectra of both **1** and **2** revealed the presence of the following residues: β-Glc*p*A (residue *a*), β-Gal*p* (residue *b*), α-Ara*p* (residue *c*), β-Fuc*p* (residue *d*), β-Qui*p* (6-deoxy-β-Glc*p*, residue *e*), α-Rha*p* (residue *f*), β-Xyl*p* (residues *g* and *h*). The HSQC spectrum confirmed the structures of the triterpene aglycon and showed the positions of the substitutions within the oligosaccharide fragments (Table 1 and Table 2). The ROESY spectra (identical for compounds **1** and **2**) disclosed the sequence of the residues in two oligosaccharides and their location at the C-3 and C-28 of the aglycon. Thus, the location of GlcA (residue *a*) at the position 3 of the triterpene was established from the presence of a correlation peak 1*a*/3Agl (Figure 2 and Figure 3). Correlation peaks 1*b*/2*a* and 1*c*/3*a* correspond to substitutions of the residue *a* by terminal *b* at the position 2 and by terminal *c* at the position 3. Esterification of the position 1 of Fuc (residue *d*) with the carboxy group of the triterpene was unambiguously shown by the high-field shift of C-1 (94.4 ppm), being indirectly confirmed with the long-range correlation peak in the ROESY spectra 16Agl/3*d*. The sequence of the other residues was disclosed from the presence of the correlation peaks 1*e*/4*d*, 1*f*/2*d*, 1*g*/4*f* and 1*g*/4*h* (Figure 2). HMBC spectra finally confirmed the structure of the aglycons and the sequence of the residues. Thus, the correlation peak 1*d*/28Agl evidenced the location of Fuc (residue *d*) as the esterified substituent at C-28 of the triterpene (Figure 4). The other inter-residue correlation peaks were in agreement with the structure of oligosaccharides established from analysis of the ROESY spectra.

Characteristic chemical shifts in the ^13^C NMR spectrum of **2** (δ_C_ 85.4 ppm for C-3 and δ_C_ 176.4 ppm for C-28 of the aglycon) evidence the bidesmosidic nature of the genin, which is glycosydated at C-3 and esterified to an oligosaccharide. The structures of both the trisaccharide and pentasaccharide fragments of compound **2** are similar to those established for compound **1**. Thus the structure of **2** was elucidated as gypsogenin 28-*O*-β-D-xylopyranosyl-(1**→**3)-β-D-xylopyranosyl-(1**→**3)-α-L-rhamnopyranosyl-(1**→**2)-[6-deoxy-β-D-glucopyranosyl-(1**→**4)]-β-D-fucopyranosyl ester 3-*O*-α-L-arabinopyranosyl-(1**→**3)-[β-D-galactopyranosyl-(1**→**2)]-β-D-glucuronopyranoside.

It should be noted that the elucidated structures of **1** and **2** are different from those reported earlier [18]. In the published structures β-D-Qui*p* (residue *e*) is located at O-2 of the β-Fuc*p* (residue *d*), and the trisaccharide moiety β-D-Xyl-(1→3)-β-D-Xyl-(1→3)-α-L-Rha is at O-4.

The acute-toxicity study of saponins **1** and **2** was carried out on albino mice. The median lethal dose (LD_50_) was determined after a single dose administered through the oral or intraperitoneal route. The obtained data show that, in the case of oral administration of the studied compounds, LD_50_ was in the range of 304 ± 55 mg/kg for compound **1** and 252 ± 57 mg/kg for compound **2** with p < 0.05 (t). In the case of intraperitoneal administration, the LD_50_ was in the range of 15.1 ± 5.6 mg/kg for compound **1** and 5.4 ± 2.8 mg/kg for compound **2** with p < 0.05 (t). The immense difference between the values of LD_50_ in oral and intraperitoneal tests evidences low or no absorption of saponins in the intestine. However, it remains to be studied whether traces or decomposition products of ingested saponins enter the blood stream through the permeable membranes of mucosal cells.

For compounds **1**, **2** and saponin from *Quillaja* bark (Sigma) as a reference compound, the study on in vitro hemolysis was carried out. The obtained data confirmed high hemolytic activity of the *Quillaja* bark saponin, which caused 100% of hemolysis at a minimal hemolytic concentration of 5.5 μg/mL. Saponins **1** and **2** exhibited much lower hemolytic activity (Table 3): 50% hemolysis was observed at concentrations 11–18 μg/mL. Hemolysis of 85–95% for compounds **1** and **2** was observed at 62.5 μg/mL, whereas saponins QS-17, 18, and 21 from *Quillaja* bark were reported [23] to cause hemolysis at concentrations of 7–25 μg/mL. These results are in good agreement with our expectations based on the factors that are known to accompany low hemolytic activity, i.e., the bidesmosidic nature of compounds **1** and **2**, the presence of glucuronic acid at C-3 of the aglycone, and the absence of a lipid moiety.

**Table 3:** Hemolytic activities (%) of compounds **1** and **2** at different concentrations. The hemolytic activities of saline and distilled water were used as minimal and maximal hemolytic controls, respectively. *n* = 3 tests. Mean *p* < 0.05 vs saline group.

Saponin	Percentage of hemolysis (concentration of saponin in saline, µg/mL)						

**1**	84.5 ± 4.4 (62.5)	76.5 ± 3.3 (25)	75.1 ± 1.6 (12.5)	8.6 ± 3 (5)	8.3 ± 2.4 (2.5)	0.7 ± 1.2 (0.5)	0 ± 1.6 (0)
**2**	93.4 ± 4.5 (62.5)	90.8 ± 4.2 (25)	15.8 ± 1.4 (12.5)	12.0 ± 1.7 (5)	11.2 ± 2.3 (2.5)	10.0 ± 1.4 (0.5)	0 ± 1.6(0)

Histamine-induced acute inflammation in the paws of the mice was used as a classical model of edema formation for the study of the anti-inflammatory activity of saponins. Two methods of saponin administration were used, namely oral (Table 4) and intraperitoneal (Table 5). In the first experiment, six groups of eight mice each were treated orally with compound **1** (20 mg/kg, 50 mg/kg), compound **2** (20 mg/kg, 50 mg/kg), indomethacin (20 mg/kg), and water (control). One hour after receiving the agents, each animal received a subcutaneous injection of 0.05 mL 0.1% histamine in the right, hind paw. The edema was measured 5 hours after the histamine injection. The anti-inflammatory effect was assessed by the decrease in the index of edema compared with the control group, which is defined as the percentage difference between the mass of the healthy and the inflamed paw, relative to the mass of the healthy paw.

**Table 4:** Anti-inflammatory effect of compounds **1** and **2** (oral administration).

parameter	dose in mg/kg					

	Water	**1**, 20	**2**, 20	**1**, 50	**2**, 50	Indomethacin, 20
Index of edema (%)	23.51 ± 5.18	20.23 ± 4.97	16.39 ± 5.49*	19.14 ± 5.79	24.57 ± 5.81***	15.79 ± 5.17*

*p < 0.05, ***p < 0.001 compared with control.

**Table 5:** Anti-inflammatory effect of compound **1** and **2** (intraperitoneal administration).

parameter	dose in mg/kg					

	Water	**1**, 1.25	**2**, 1.25	**1**, 2.5	**2**, 2.5	Indomethacin, 20
Index of edema (%)	28.22 ± 6.07	24.06 ± 9.11	17.32 ± 7.6**	14.88 ± 5.17***	12.28 ± 3.94***	19.43 ± 6.7*

*p < 0.05, ** p < 0.01, ***p < 0.001 compared with control.

In general, the anti-inflammatory effect of saponins **1** and **2** given intraperitoneally was dose-dependent, whereas that in the experiment with oral administration was not. Within the experiment based on oral administration, compound **1** did not show any reliable anti-inflammatory action. Data given in Table 5 evidences the more pronounced anti-inflammatory properties of compound **2** as compared to compound **1** in the experiment based on intraperitoneal administration.

The influence of saponins **1** and **2** on the vessel endothelium was assessed via determination of interleukin-6 (IL-6) production in primary human umbilical vein endothelial cells (HUVEC). These cells were found to express various pattern-recognizing receptors (PRRs), including TLR4 [24], and can produce pro-inflammatory cytokines, including IL-6, IL-8 and IL-1β, upon stimulation with bacterial and viral components, such as lipopolysaccharides (LPS) and double-stranded RNA (dsRNA) [25].

**Figure 5:**
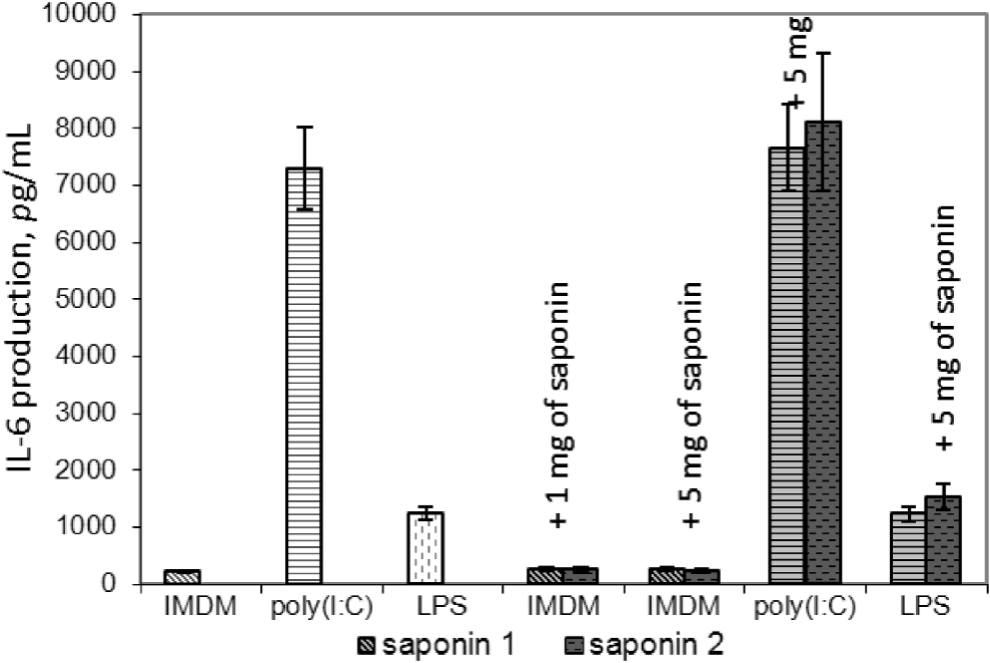
IL-6 production of primary endotheliocytes in the presence of compounds **1** and **2**. Error bars represent the standard deviation in each point.

HUVEC were cultivated in the presence of saponins **1** and **2** at nontoxic concentrations, i.e., 1 and 5 μg/mL, as determined by EZ4U test, and reference compounds LPS from *E. coli* and dsRNA analogue poly(I:C). Measurement of IL-6 production did not show any effect of saponins **1** and **2** on IL-6 secretion, either by testing intact endothelial cells or cells pre-stimulated with LPS or poly(I:C) (Figure 5), indicating that at nontoxic concentrations saponins are not able to induce an innate immune response in endothelium. Thus we can conclude that compounds **1** and **2** are not prone to cause inflammation of the vessel endothelium.

To estimate the adjuvant properties of compounds **1** and **2** a series of test immunizations was carried out by using the synthetic vaccine neoglycoconjugate α-NeuAc-(2**→**3)-β-Gal*p*-(1**→**4)-β-Glc*p*-KLH (3’SL-KLH) on the basis of 3’-sialyllactoside (3’SL) ligands and keyhole limpet hemocyanin (KLH) protein carrier [26]. Four groups of mice were immunized with 40 µg of 3’SL-KLH together with 50 µg of compound **1** or **2**, or saponin from *Quillaja* bark, or without an adjuvant, and the specific anti-3’SL IgM and IgG responses were evaluated. For saponins **1** and **2**, a significant specific response was observed in comparison with the control vaccine formulation with antigen alone (Table 6). High serum titers of IgM and IgG antibodies were registered in the vaccination with compound **1** as adjuvant, though the IgG level did not achieve the level measured in the experiment with saponin from *Quillaja* bark. Titers of those antibodies in the experiment with saponin **2** were rather low. We can conclude, that in combination with 3’SL-KLH, antigen compound **1** showed significant adjuvant properties and, hence, can be considered as a prospective component of vaccine formulations.

**Table 6:** Serum titer data for antisera against 3’SL-polyacrylamide cover antigen obtained by immunization with 3’SL-KLH neoglycoconjugate with adjuvants **1**, **2**, and saponin from *Quillaja* bark, or without an adjuvant.

adjuvant	IgM	IgG

no adjuvant	1/1600	1/800
saponin **1**	1/102400	1/51200
saponin **2**	1/12800	1/6400
saponin from *Quillaja* bark	1/25600	1/204800

**Figure 6:**
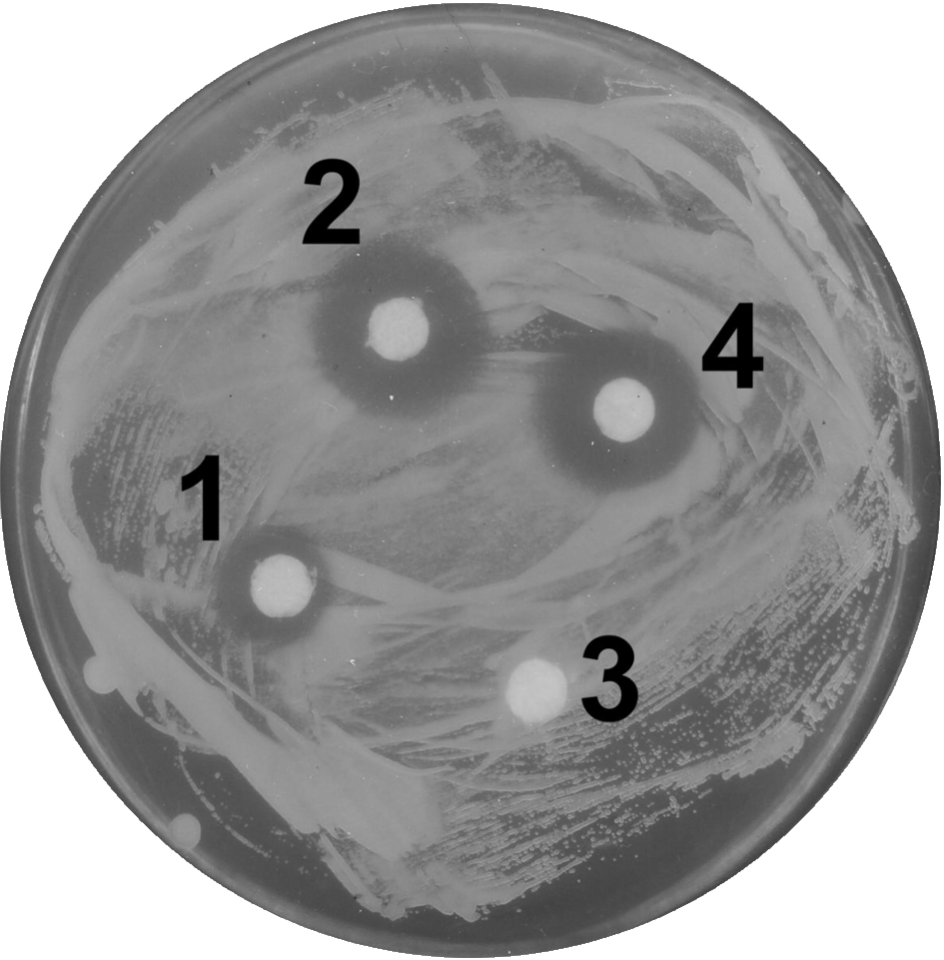
Growth inhibition zones for *F. neoformans* IGC 3957 in the presence of compounds **1** and **2** at pH 4.0. Compound **1**: (1) 0.25 mg/disc, (2) 0.5 mg/disc; compound **2**: (3) 0.25 mg/disc, (4) 0.5 mg/disc.

As many saponins are known to exhibit antifungal activities [2], we examined the ability of compounds **1** and **2** to suppress the proliferation of four test cultures: Basidiomycetous yeasts *Cryptococcus terreus*, *Filobasidiella neoformans*, and ascomycetous yeasts *Saccharomyces cerevisiae* and *Саndida albicans*. The data in Table 7 demonstrate that compounds **1** and **2** exhibit antifungal activity against both ascomycetous and basidiomycetous yeasts, including the medically important *C. albicans* and *F. neoformans.* Saponins are known to be more effective against basidiomycetous yeast and at acidic pH act similarly to natural detergents, such as cellobiose lipids [27]. Growth inhibition experiments showed (Figure 6, Table 7), that the lower pH 4.0 favored antifungal activity of saponins **1** and **2**. At pH 7.0 neither compound **1** nor **2** inhibited the growth of *C. albicans* and *F. neoformans*. However, at pH 4.0 both saponins exhibited suppressing properties against these two strains. Notably, compound **2** was totally inactive against *S. cerevisiae* at both pH values. The liquid medium test involving *F. neoformans* showed only slight differences between saponins **1** and **2** (Table 8).

**Table 7:** Growth inhibition zones (Figure 6) of *C. terreus* (*C.t.*), *S. cerevisiae* (*S.c.*), *F. neoformans* (*F.n.*) and *C. albicans* (*C.a.*) in the presence of compounds **1** and **2** at pH 7.0 and 4.0.

pH	compound	amount,	diameter of growth inhibition zone, mm			
			
		mg/disc	*C.t.*	*S.c.*	*F.n.*	*C.a.*

7.0	**1**	1.0	n.d.^a^	n.d.	0	0
		0.5	12	10	0	0
		0.25	7	0	0	0
		0.1	0	0	0	0
		0.05	0	0	0	0
	**2**	1.0	n.d.	n.d.	0	0
		0.5	0	0	0	0
		0.25	0	0	0	0
		0.1	0	0	n.d.	n.d.
		0.05	0	0	n.d.	n.d.

4.0	**1**	1.0	n.d.	n.d.	n.d.	14
		0.5	18	10	15	10
		0.25	14	5	10	0
		0.1	10	0	n.d.	n.d.
		0.05	0	0	n.d.	n.d.
	**2**	1.0	n.d.	n.d.	n.d.	14
		0.5	13	0	15	7
		0.25	10	0	0	0
		0.1	10	0	n.d.	n.d.
		0.05	0	0	n.d.	n.d.

^a^n.d. not determined.

**Table 8:** Viability (%) of *F. neoformans* IGC 3957 treated with compounds **1** and **2** at pH 4.0.

	concentration, mg/mL			
	
compound	0	0.49	0.97	1.87

**1**	100	66 ± 5.6	11 ± 0.1	4 ± 0.3
**2**	100	93 ± 7.2	18 ± 1.8	6 ± 0.5

## Conclusion

Thus, the structures of two easily available novel saponins **1** and **2** were elucidated, and they were shown to exhibit low hemolytic activity, low oral and intraperitoneal toxicity, and an inability to induce inflammation in the vessel endothelium. Meanwhile, compounds **1** and **2** exhibited prominent immune-stimulating properties and can be considered as a prospective adjuvant in combination with KLH-based neoglycoconjugates. The antifungal activity of saponins **1** and **2** was also examined on four yeast species.

## Experimental

**General experimental procedures.** Optical rotation values were measured on a JASCO DIP-360 polarimeter at 22 ± 2 °C. NMR spectra were recorded on Bruker DRX-500 and Bruker AM-300 instruments in D_2_O/pyridine-*d*_5_ with TMS as internal reference. High-resolution mass spectra (HRMS) were measured on a Bruker micrоTOF II instrument by using electrospray ionization (ESI) [28]. The measurements were done in a positive-ion mode (interface capillary voltage: 4500 V) or in a negative-ion mode (3200 V); mass range from *m*/*z* 50–3000 Da; external or internal calibration was achieved with electrospray calibrant solution (Fluka). A syringe injection was used for solutions in acetonitrile, methanol, or water (flow rate 3 μL/min). Nitrogen was applied as a dry gas; the interface temperature was set to 180 °C. High-performance liquid chromatography (HPLC) was carried out on a C18 reversed-phase column (Ascentis C18, 5 μm, 250 × 21.2 mm, 65% MeOH, 35% of 0.05 M aq. solution of NH_4_HCO_3_) with the use of a UV detector at 210 nm. Analysis of purity was carried out on a C18 reversed-phase column (IBM C18, 5 μm, 250 × 4.5 mm) with eluent and detection as described above.

**Statistical analysis.** Statistical significance was determined by the Student’s *t*-test. *P* values less than 0.05 were considered to be significant. The Kaplan–Meier and one-way ANOVA analysis was used to establish statistical significance for the in vivo experiments.

**Plant material.** The roots of *A. gypsophiloides* Rgl*.* were collected in September 2005 on a mountainside in the Chimkent region, Kazakhstan and were identified by Prof. P. G. Gorovoi (G.B. Elyakov Pacific Institute of Bioorganic Chemistry, Far Eastern Branch, Russian Academy of Sciences). A herbarium specimen (herbarium no. 03025) has been deposited at the Herbarium of Novosibirsk Botanical Garden; Russia. Saponin from *Quillaja* bark was purchased from Sigma–Aldrich (S4521) and used without further purification.

**Extraction and isolation.** Dried and finely powdered roots of *A. gypsophiloides* (490 g) were heated in methanol (5 × 800 mL) under reflux for 10 h, filtered, and concentrated to yield the extract (86.5 g, 17.6%). This extract was dissolved in methanol (87 mL) and precipitated by the addition of acetone (750 mL). The resulting precipitate was filtered and dried in a vacuum desiccator over dry KOH (fraction A, 14.5 g, 3.0%). The mother liquid was concentrated and subjected to silica gel column chromatography; elution with a mixture of CHCl_3_/MeOH/H_2_O 130:70:15 gave 29.3 g of an amorphous residue, which was dissolved in 500 mL of 2-propanol and 0.2 mL of acetic acid and evaporated under reduced pressure to remove residual water. The residue was triturated with 20 mL of methanol and the slurry was diluted with acetone (160 mL). The formed precipitate was filtered and dried to give 22.2 g of total glycosides (fraction B). Combined fractions A and B (36.7 g) were subjected to preparative HPLC (Ascentis C18, 5 μm, 250 × 21.2 mm) using a mixture of 65% methanol and 35% 0.05 M aq. solution of NH_4_HCO_3_ to yield 18.35 g of compound **1** (3.7% starting from the root powder) and 15.42 g of compound **2** (3.1% starting from the root powder) as ammonium salts. The purity of **1** and **2** was assessed by analytical C18 reversed-phase HPLC and varied in a range of 97–99%.

**Monosaccharide analyses.** Compounds **1** and **2** were hydrolyzed with 2 M CF_3_CO_2_H (120 °C, 2 h) and the absolute configurations of the monosaccharides were determined by GLC of acetylated (*S*)-(+)-2-octyl glycosides according to the published method [29]. GLC was performed using an Agilent 7820A chromatograph equipped with an HP-5 fused silica column (0.25 mm × 30 m) using a temperature program of 160 °C to 290 °C (7 °C min^−1^).

**3-*O*-[β-D-Galactopyranosyl-(1→2)-[α-L-arabinopyranosyl-(1→3)]-β-D-glucuronopyranosyl]quillaic acid 28-β-D-xylopyranosyl-(1→3)-β-D-xylopyranosyl-(1→3)-α-L-rhamnopyranosyl-(1→2)-[6-deoxy-β-D-glucopyranosyl-(1→4)]-β-D-fucopyranosyl ester (1):** white amorphous solid; [α]^20^_D_ −5.0 (*с* 1, Н_2_О); ^1^H and ^13^C NMR data, see Table 1 and Table 2; HRMS–ESI^+^ (*m*/*z*): [M + Na]^+^ calcd for C_75_H_118_O_40_Na, 1681.7097; found, 1681.7071.

**3-*O*-[β-D-Galactopyranosyl-(1→2)-[α-L-arabinopyranosyl-(1→3)]-β-D-glucuronopyranosyl]gypsogenin 28-β-D-xylopyranosyl-(1→3)-β-D-xylopyranosyl-(1→3)-α-L-rhamnopyranosyl-(1→2)-[6-deoxy-β-D-glucopyranosyl-(1→4)]-β-D-fucopyranosyl ester (2):** white amorphous solid; [α]^20^_D_ 5.0 (*с* 1, Н_2_О); ^1^H and ^13^C NMR data, see Table 1 and Table 2; HRMS–ESI^+^ (*m*/*z*): [M + Na]^+^ calcd for С_75_H_118_O_39_Na, 1665.7148; found, 1665.7181.

**Acute-toxicity assay.** Albino, nonbreeding, sexually mature male mice from SPF-vivarium of SB RAS weighing 20–25 g were used in the test. All research involving laboratory animals was carried out in accordance with The Guidelines for the Care and Use of Laboratory Animals. Mice were housed in appropriate caging facilities and allowed food and water ad libitum.

Oral route: The experiment involved 50 male mice. Animals were selected at random and divided into five groups, each consisting of 10 mice. The control group received only pelleted food and water. The other four groups received pelleted food and water along with varying doses of compounds **1** or **2**, at either 50.0 mg/kg, 100.0 mg/kg, 250.0 mg/kg or 500.0 mg/kg. Saponins were given by single oral gavage in the prescribed doses by using a feeding cannula. The acute LD_50_ toxicity of saponins **1** and **2** was calculated on the basis of the mortality data collected within seven days by using Probit Analysis 1.0 software with p < 0.05.

Intraperitoneal route*.* The experiment involved 50 male mice. Animals were selected at random and divided into five groups, each consisting of 10 mice. The control group received only pelleted food and water. The other four groups received pelleted food and water and were inoculated once intraperitoneally with solutions of varying doses of saponins **1** or **2**, at either 0.50 mg/kg, 5.0 mg/kg, 10 mg/kg or 50.0 mg/kg in saline. The acute LD_50_ toxicity of saponins **1** and **2** was calculated on the basis of the mortality data collected within seven days using Probit Analysis 1.0 software with p < 0.05.

**Hemolysis assay.** Red blood cells were obtained from Wistar sexually mature rats of both sexes from SPF-vivarium of SB RAS, weighing 200–250 g. Blood was collected from neck vessels in standard plastic tubes containing 3.8% solution of sodium citrate. Aliquots of 7 mL of citrated blood (volume ratio of blood to sodium citrate 9:1) were washed with sterile non-pyrogenic saline (0.89% sodium chloride). Washing was performed by adding an equal volume of saline solution to an aliquot of citrated blood and subsequent centrifugation at 180 g for 5 min, after which the supernatant was discarded, and the procedure repeated three times. Harvested erythrocytes were diluted with saline to obtain a suspension of 0.5% hematocrit. Samples containing 0.5 mL of cell suspension were mixed with 0.5 mL of saline solutions (145 mM, isotonic conditions) containing the investigated saponins in concentrations of 5, 10, 15, 25, 50, 75, 100, 250, 500 μg/mL. Samples were stirred continuously for 30 min at 37 °C and then centrifuged at 70*g* for 10 min. The content of free hemoglobin in the supernatant was measured by spectrophotometric analysis at a wavelength of 412 nm (spectrophotometer Cary 50, Varian). Hemoglobin concentration in the supernatant was expressed as a percentage of hemoglobin concentration in the supernatant of cells, which were totally hemolysed by the addition of distilled water. The absorbance of samples with 0% hemolysis was registered for samples with saline and used as a blank measurement. The degree of hemolysis, depending on the concentration of saponin was calculated by using Probit Analysis 1.0 software.

**Anti-inflammatory activity.** Albino, nonbreeding, sexually mature male mice from SPF-vivarium of SB RAS weighing 20–25 g were used in the test. All research involving laboratory animals was carried out in accordance with The Guidelines for the Care and Use of Laboratory Animals. Mice were housed in appropriate caging facilities and allowed food and water ad libitum.

Oral route: Six groups of eight mice were treated orally with saponin **1** (20 mg/kg, 50 mg/kg), saponin **2** (20 mg/kg, 50 mg/kg), and indomethacin (20 mg/kg) in Twin-80 water solution (control). One hour after receiving the agents, each animal received a subcutaneous injection of 0.05 mL 0.1% histamine in the right hind paw. The edema was measured 5 h after the histamine injection as the difference in weight between the paw that was administered histamine, and a healthy paw. The anti-inflammatory effect was assessed by the decrease in index of edema compared with the control group. The index of edema is defined as the ratio of the difference between the masses of the inflamed and healthy paws to the mass of the healthy paw in percent: (Mi − Mh) / Mh × 100%; Mi: mass of inflamed paw, Mh: mass of healthy paw. A probability of p < 0.05 was considered significant.

Intraperitoneal route: Six groups of eight mice were treated intraperitoneally with saponin **1** (1.25 mg/kg, 2.5 mg/kg), saponin **2** (1.25 mg/kg, 2.5 mg/kg) and indomethacin orally (20 mg/kg) in Twin-80 water solution (control). One hour after receiving the agents, each animal received a subcutaneous injection of 0.05 mL 0.1% histamine in the right hind paw. The edema was measured 5 h after the histamine injection as the difference in weight between the paw that was administered histamine, and a healthy paw. The anti-inflammatory effect was assessed as described above.

### Cytotoxicity, cell proliferation test and endotoxin test

**Cell cultures:** Primary endothelial cells were obtained from the human umbilical vein [30] and cultivated in Iscove's Modified Dulbecco's Medium (IMDM, Gibco, USA, 42200-014) with 10% fetal bovine serum (FBS) (Gibco, USA, 10106), 100 μg/mL streptomycin, and 100 μg/mL penicillin (IMDM-FBS) at 37 °С with 5% СО_2_. Primary endotheliocytes were seeded on 0.5% gelatin precoated (Sigma, USA, G-2500) culture dishes or microplate wells and were detached with 0.1% collagenase solution (Gibco, USA, 17104-019). To examine the cytotoxic and pro-inflammatory effects of saponins, primary endotheliocytes were seeded at a density of 7 × 10^3^ cells per well of a 48-well microplate. Sixteen hours after seeding, cells were washed with IMDM, and 200 μL (48-well plate) of IMDM-FBS with saponins (from 1 to 100μg/mL) was added and cultivated for a subsequent 24 h.

**EZ4U test:** After incubation with saponins, cells were washed with IMDM-FBS and cultivated with 100 μL of fresh IMDM-FBS containing components of EZ4U kit (EZ4U, Biomedica, Austria) for a subsequent 24 h. After incubation, the culture supernatants were transferred into a 96-well plate and the optical density was registered in a Multiscan plate reader at 450 nm (SDB NP Puschino, Russia). All experiments were performed in triplicate.

**LAL test:** All components contacting with cells were tested for endotoxin contamination by using the LAL gel clot test as recommended by the producers (Associate of Cape Cod Incorporated, USA).

**IL-6 release assay:** Primary endothelicytes were incubated in the IMDM-FBS with saponins at noncytotoxic concentrations (1 and 5 μg/mL) in the absence or in presence of LPS (100 ng/mL) or poly-(I:C) (Sigma, USA, P9582, 100 μg/mL) at 37 °С, 5% СО_2_ for 24 h. For positive control, cells were incubated in the presence of 100 μg/mL of poly-(I:C) or 100 ng/mL of LPS from *E. coli* (LPS) (Sigma, USA, L2755). Cells were incubated in IMDM-FBS for 24 h, culture medium was removed, and cells were centrifuged at 1500*g* and preserved at −20 °С. The IL-6 concentration in the samples was determined by using a commercial ELISA kit (Vector-Best, Russia, A-8768) according to the protocol suggested by the manufacturer. All experiments were performed in triplicate.

**Studies of adjuvant activity:** *Animals.* Female Swiss mice (eight weeks old) of the C57B1/6J breed were purchased from the “Stolbovaya”, Russia, and rodent laboratory chow and tap water were provided ad libitum. Mice were maintained under a controlled temperature (22 ± 2 °С) and humidity under a 12/12 h light/dark cycle. All the procedures were carried out in strict accordance with the International Legislation on the Use and Care of Laboratory Animals.

**Glycoconjugate vaccine preparation:** Conjugate (3’SL-KLH [28]) of α-NeuAc-(2**→**3)-β-Gal*p*-(1**→**4)-β-Glc*p* ligands with keyhole limpet hemocyanin carrier (KLH), bearing 5% mass of indicated carbohydrate, was used as an immunogen, and saponins **1** and **2** and saponin from *Quillaja* bark were used as adjuvants. All samples were filtered through 0.22 μm Micropore® filters and kept at 4 °C prior to use.

**Immunization:** Four groups of seven mice each were immunized intramuscularly thrice, on days 0, 14, and 84 with a mixture of 40 μg of conjugate and 50 μg of different saponins (or without adjuvant for the control group) in PBS as a vehicle in a total vaccine volume of 200 μL.

**ELISA:** Sera from inoculated mice were collected on day 91 post-inoculation (p.v.) of the first dose of vaccine and pooled. The titers for IgG and IgM against α-NeuAc-(2**→**3)-β-Gal*p*-(1**→**4)-β-Glc*p* were determined in an indirect ELISA as previously described [31]. ELISA plates (96-well, Nunc Maxisorp) were coated with a cover polyacrylamide antigen [28] with α-NeuAc-(2**→**3)-β-Gal*p*-(1**→**4)-β-Glc*p* moieties. Coating was performed with a 10 μg/mL in 0.1 M bicarbonate buffer solution at 4 °C overnight. Wells were washed three times with PBS containing 0.05% Tween 20 (PBS-T) and blocked with 1% solution of HSA in PBS-T. Diluted sera (100 µL) collected from the mice in PBS-T was added to wells and incubated overnight at 4 °C. The plates were washed three times with PBS-T, and goat anti-mouse IgG or IgM peroxidase conjugate (Jackson Immuno Research) in 1:1500 dilution (PBS-T) was added to the wells. Plates were then incubated for 1 h at 37 °C and washed, and substrate (*o*-phenylenediamine, 0.4 mg/mL in 0.1 M phosphate-citrate buffer with 0.0013% H_2_O_2_) was added to each well. Plates were then incubated for 25 min at 37 °C, after which the reaction was terminated by adding 50 μL per well of 2 N H_2_SO_4_. The optical density (OD) was measured in an ELISA plate reader at 492 nm. Data were expressed as the mean OD value of the samples minus the mean OD value of the control wells. The value of the OD for the control group was less than 0.1 (dilution 1/100 and more). Antibody levels in the sera of all samples were higher than the control (*p* < 0.05).

### Antifungal activity

Strains and culture conditions: The basidiomycetous yeasts *Cryptococcus terreus* VKM Y-2253 (All-Russian Collection of Microorganisms), *Filobasidiella neoformans* IGC 3957 (Portuguese Yeast Culture Collection, Centro de Biologia, Portugal), and ascomycetous yeasts *Saccharomyces cerevisiae* VKM Y-1173 (All-Russian Collection of Microorganisms), *Candida albicans* JCM 1542 (Japan Collection of Microorganisms) were used as test-cultures. Strains were maintained on malt agar slants at 5 °C. *S. cerevisiae* VKM Y-1173 was grown in YPD medium containing glucose (20 g/L), yeast extract (10 g/L) and peptone (20 g/L; Sigma, USA) under shaking at 30 °C for 24 h. *Candida albicans*, *Filobasidiella neoformans*, and *Cryptococcus terreus* were grown under the same conditions for 48 h in YPD medium containing glucose (20 g/L), yeast extract (4 g/L), peptone (5 g/L).

**Antifungal activity assay:** Sterile 5 mm diameter glass microfiber filter discs GF/A (Whatman, UK) were placed onto the surface of a solid medium in Petri dishes inoculated with test cultures. Two media were used: YPD containing 0.5% glucose, 0.2% yeast extract, 0.25% peptone, 2% agar, and 0.04 M citrate–phosphate buffer (pH 4.0), and YPD with 2% agar (pH 7.0). Aliquots of saponin solutions in deionized water were pipetted onto discs. The plates were incubated at 24 °C for 2–3 days until growth of the lawn strain appeared, and the diameters of the growth inhibition zones were measured. For the assay of cell viability the suspension of the cells *F. neoformans* (3 × 10^6^ cells mL^−1^) was treated with saponins in 0.01 M citrate buffer (pH 4.0) at room temperature for 1 h. Thereafter, the samples were diluted by the same buffer and inoculated on YPD agar. Three days later, the number of colonies was determined. The samples without saponins were used as a control. All experiments were repeated twice. Fresh solutions of saponins in deionized water were used.
